# Clinical Validation of the Onclarity Assay After Assay Migration to the High-Throughput COR Instrument Using SurePath Screening Samples From the Danish Cervical Cancer Screening Program

**DOI:** 10.1093/ajcp/aqab138

**Published:** 2021-09-21

**Authors:** Ditte Møller Ejegod, Helle Pedersen, Birgitte Tønnes Pedersen, Christine Monceyron Jonassen, Agnes Kathrine Lie, Laila Solhaug Hulleberg, Marc Arbyn, Jesper Bonde

**Affiliations:** 1 Molecular Pathology Laboratory, Department of Pathology, Copenhagen University Hospital AHH-Hvidovre, Hvidovre, Denmark; 2 Center for Laboratory Medicine, Østfold Hospital Trust, Grålum, Norway; 3 Unit of Cancer Epidemiology, Belgian Cancer Centre, Sciensano, Brussels, Belgium

**Keywords:** HPV assays, Genotyping, Onclarity, International validation, High throughput

## Abstract

**Objectives:**

This study presents the clinical assessment of the Onclarity HPV Assay (Becton Dickinson) on the novel COR high-throughput instrument (Becton Dickinson) using the international guidelines in a routine setting.

**Methods:**

Screening samples collected in BD SurePath from women aged 30 years and older were used in this validation. Noninferiority of the Onclarity HPV Assay on the COR instrument (Onclarity-COR) was assessed with the comparator assay glycoprotein 5–positive (GP5+)/6+ enzyme immunoassay (GP-EIA) for clinical sensitivity on 122 cervical intraepithelial neoplasia 2 and greater samples. Specificity was assessed using 887 samples with twice-normal cytology. Inter- and intralaboratory reproducibility analysis was assessed using 525 samples. Finally, a time-and-motion study was performed to evaluate COR instrument performance characteristics.

**Results:**

The Onclarity-COR was noninferior to the GP-EIA for both sensitivity (*P* = .0016) and specificity (*P* < .0001). The intralaboratory reproducibility was 98.3% (κ = 0.96), and interlaboratory agreement was 98.5 % (κ = 0.96). The daily hands-on time for the COR instrument was 58 minutes, and walk-away time was 7 hours, 2 minutes per 8-hour day shift.

**Conclusions:**

The Onclarity-COR instrument fulfills international validation criteria on sensitivity, specificity, and laboratory reproducibility. The Onclarity assay’s extended genotyping capability, together with its high-throughput characteristics, makes the COR instrument an excellent candidate for use in human papillomavirus primary cervical cancer screening.

Key Points

- We evaluated the BD COR high-throughput platform combined with the BD Onclarity HPV assay.
- Successful clinical validation was compliant with the international guidelines for human papillomavirus (HPV) assays for cervical cancer screening.
- The BD COR high-throughput platform allows for unprecedented laboratory HPV test production with limited operator interaction requirements.

## Introduction

Cervical cancer screening is transitioning worldwide from cytology toward primary human papillomavirus (HPV) screening. At the same time, there is a paradigm shift toward risk-based guidelines using clinical action thresholds.^1^ A trend in cervical cancer screening programs is a continued consolidation process toward larger, centralized laboratories with unprecedented high throughput. This shift necessitates validation of existing HPV assays and novel high-throughput HPV test instruments using well-defined clinical assay performance specifications compliant with the international criteria for HPV tests used in cervical cancer screening.^2^ The migration of an already-validated HPV test to a new high-throughput instrument platform can result in changed test characteristics. Validation of the new combination of assay and instrument is therefore as important as the original validation of the assay itself, especially if the new high-throughput platform entails different sample processing from the previous instrument. Operator interaction (hands-on time), workflow, and system maintenance requirements are also important determinants for the clinical performance of fully automated high-throughput HPV instruments, and these elements can differ considerably between automated systems.^3,4^

Commercially available high-throughput HPV instruments are only now being introduced and validated. The cobas HPV Test on the cobas 6800/8800 instruments from Roche Diagnostics,^4-6^ the Alinity instrument from Abbott,^7,8^ and the COR HPV instrument from BD Diagnostics are all branded on sample turnaround numbers surpassing their predecessors: the Roche Diagnostics cobas 4800,^9^ the Abbott RealTime High Risk HPV Assay,^10^ and the BD Viper LT system,^11,12^ respectively.

Besides the move toward larger instruments for existing assays, a change toward the use of HPV genotype information in management algorithms is defining cervical cancer screening. The 2019 ASCCP guidelines describe a paradigm shift to risk-based guidelines using clinical action thresholds.^1,13^ Here, HPV genotyping plays a role in the management of index screening samples but also, when combined over time with subsequent follow-up samples or multiple screening rounds, enables clinicians to assess HPV persistence vs new infections as an integrated element in the evaluation of risk of disease and subsequent management.

The BD Onclarity HPV Assay is an extended genotyping assay that can detect 6 individual HPV genotypes and 8 genotypes in 3 groups (individual: 16, 18, 31, 45, 51, 52; Groups: 33/58, 56/59/66, 35/39/68). The Onclarity HPV Assay has been validated according to the international guidelines for samples collected in both ThinPrep and SurePath liquid-based cytology media in several studies.^11,12,14-16^ It is US Food and Drug Administration (FDA) approved for clinical cervical cancer screening using the Viper LT instrument.^17-19^ The newly developed, fully automated high-throughput BD COR instrument runs the Onclarity HPV Assay (Onclarity-COR) with identical chemistry, aspiration, and transfer volumes as on the well-validated Viper LT instrument (Onclarity-Viper). The COR system received CE Mark approval in 2019, and a premarket approval supplement was submitted to the FDA in 2020. The COR instrument consists of 2 interconnected units: a processing unit (PX) and an analytical unit (GX). SurePath or ThinPrep vials are loaded directly into the COR system, which handles the scanning, aliquoting, preheat treatment, DNA extraction, and real-time polymerase chain reaction (RT-PCR) analysis in a fully automated workflow that performs all preanalytic and analytic assay testing in a single system.

Here, we validate the Onclarity HPV assay after migration from the Viper LT instrument to the COR high-throughput HPV instrument using SurePath screening samples from women aged 30 years and older who participated in the organized cervical cancer screening program in Denmark. The validation is performed using the samples from the fourth installment of the VALGENT validation study,^20,21^ where clinical sensitivity and specificity were evaluated against glycoprotein 5–positive (GP5+)/6+ enzyme immunoassay (GP-EIA), accepted as a standard comparator assay. Furthermore, the validation panel was complemented with an independently collected reproducibility panel in concordance with international validation guidelines.^2^ A detailed genotype concordance analysis between Onclarity-COR and Onclarity-Viper was performed as well. Finally, a time-and-motion study was conducted to assess the laboratory performance of the COR instrument in a routine 8-hour day setting.

## Material and Methods

### Sample Collection and Histologic Follow-up

Sample collection, processing, cytology, and histology procedures have previously been described in detail.^20^ In short, the VALGENT4 panel was collected at the Department of Pathology, Hvidovre Hospital, Denmark (parent laboratory) in 2016 and consisted of 2 populations: 998 consecutive screening samples from routinely screened women (screening population; average age, 42.8 years [range, 30-59 years]) consisting of 947 samples negative for intraepithelial lesions or malignancy (NILM); 6 with abnormal squamous cells of undetermined significance (ASCUS); 21 with low-grade squamous intraepithelial lesions (LSILs); and 24 with high-grade SILs (HSILs), atypical glandular cells, atypical cells—cannot exclude HSIL, and adenocarcinoma in situ  Table 1 . The second population was a disease-enriched cohort of 100 ASCUS, 100 LSIL, and 97 HSIL cytology samples (the enriched population; average age, 40.4 years [range, 30-59 years]). Histology was assessed from the Danish Patobank 33 months (range, 32-35 months) after baseline and revealed 122 cervical intraepithelial neoplasias (CINs) 2 or above, with the majority originating from the enriched population. In addition, 887 samples from women with consecutive cytology NILM at baseline and 12 to 24 months prior represented women without disease. Performance on the COR instrument was compared with previously obtained results on the Viper LT instrument.^14^

**Table 1:** Characteristics of the Study Population and Human Papillomavirus Prevalence by Onclarity-COR, Onclarity-Viper, and GP-EIA

		Onclarity-COR	Onclarity-Viper	GP-EIA Assay
	Total	hrHPV-Positive, No. (%)	hrHPV-Positive, No. (%)	hrHPV-Positive, No. (%)
All	1,295	369 (28.3)	369 (28.4)	396 (30.6 )
Age, y				
30-39	531	194 (36.5)	193 (36.3)	202 (38.0)
40-49	519	124 (23.9)	130 (25.0)	136 (26.2)
50-59	245	51 (20.8)	51 (20.8)	58 (23.7)
Cytology				
Normal	947	75 (7.9)	78 (8.2)	105 (11.1)
ASCUS	106	100 (94.3)	103 (97.2)	97 (91.5)
LSIL	121	88 (72.7)	88 (72.7)	88 (72.7)
HSIL	106	95 (89.6)	94 (88.7)	96 (90.6)
AGC/ASCH/AIS	15	11 (73.3)	11 (73.3)	10 (66.7)
Histologic follow-up				
No biopsy	946	108 (11.4)	111 (11.7)	139 (14.7)
NILM	154	80 (51.9)	82 (53.2)	78 (50.6)
CIN1	73	66 (90.4)	67 (91.8)	66 (90.4)
CIN2	39	35 (89.7)	34 (87.2)	35 (89.7)
CIN3	75	72 (96.0)	72 (96.0)	70 (93.3)
Carcinoma	8	8 (100)	8 (100)	8 (100)
≥CIN2	122	115 (94.3)	114 (93.4)	113 (92.6)
≥CIN3	83	80 (96.4)	80 (96.4)	78 (94.0)
2×NILM^1^	887	66 (7.4)	70 (7.9)	95 (10.7)

2×NILM, NILM at baseline and 12-24 months prior; AGC, atypical glandular cell; AIS, adenocarcinoma in situ; ASCH, atypical cells—cannot exclude HSIL; ASCUS, abnormal squamous cells of undetermined significance; CIN, cervical intraepithelial neoplasia; GP-EIA, glycoprotein 5–positive/6–positive enzyme immunoassay; hrHPV, high-risk human papillomavirus; HSIL, high-grade squamous intraepithelial lesion; LSIL, low-grade squamous intraepithelial lesion; NILM, negative for intraepithelial lesions or malignancy; Onclarity-COR, Onclarity HPV Assay on the COR instrument; Onclarity-Viper, Onclarity HPV Assay on the Viper LT instrument.

The reproducibility agreement panel contained 525 samples collected from routine samples tested with the Onclarity HPV assay on the Viper LT instrument from Danish women going for screening, with a predefined split of 32% HPV-positive and 68% HPV-negative samples, as stipulated by the international guidelines^2^—in total, 169 positives and 355 negatives. In total, 525 samples were tested twice for intralaboratory agreement at the parent laboratory in Copenhagen. The original SurePath vial was tested on the COR instrument twice on 2 separate runs (range, 0-6 days). For interlaboratory agreement, an aliquot of the samples, preprocessed at the parent laboratory from the original vial to instrument-compliant molecular (M) tubes, was shipped to Østfold Hospital Trust, Norway, and tested once. Only samples valid on all 3 runs were included in the analysis.

### Onclarity Testing on the COR Instrument

The Onclarity assay is an RT-PCR assay with extended genotyping for 9 genotype readouts (16, 18, 31, 45, 51, 52, 33/58, 35/39/68, and 56/59/66). The Onclarity assay on the COR instrument uses the same chemistry and sample aspiration and transfer volumes as the Onclarity assay on the Viper LT instrument.^11,14,22^ The COR system has a flexible, modular design and consists of a centralized preanalytical PX module that performs all primary specimen conversions, including uncapping/capping, vortexing, aliquoting, heating (as needed), specimen holding (as needed), and control rehydration.

The processed specimens and controls are then sent to the analytic GX module, which is equipped with full assay automation (without user intervention). Depending on the particular needs of the laboratory, the preanalytical PX module can be connected with up to 2 independent GX modules, thus offering flexible options dependent on capacity needs. We evaluated the performance of the PX module paired with a single GX unit. Onclarity testing on the COR instrument was performed in 2019, with mean time from sample reception at the laboratory to testing of 1,185 days (range, 1176-1205 days).

### Comparator Testing

The GP-EIA was used as the standard comparator for clinical accuracy of the sensitivity detection of ≥CIN2 or ≥CIN3 and the specificity of <CIN1 using the Onclarity assay performed on the COR instrument. The high-risk HPV (hrHPV) GP-EIA assay has pooled detection of 14 hrHPV types (16, 18, 31, 33, 35, 39, 45, 51, 52, 56, 58, 59, 66, 68), and the GP-EIA testing was performed as part of the VALGENT4 study described previously.^20^

### Onclarity Testing on the Viper LT Instrument

Onclarity testing was performed on the Viper LT instrument according to the manufacturer’s recommendations and as described previously.^14^ Testing was performed in 2016, with mean time from sample reception at the laboratory to testing of 28 days (range, 2-70 days). The samples were stored refrigerated during this period and were subsequently stored at −20°C before COR testing.

### Time-and-Motion Study

A time-and-motion study of the COR instrument workflow was performed. For 1 working week, 330 samples were loaded into the COR daily for a single-shift workflow. Time points were assessed on each of the 5 days for (1) system startup, (2) daily maintenance, (3) weekly maintenance, (4) reagent loading, and (5) sample loading. Hands-on time and time to result for each day were registered.

Two daily interactions were required with the instrument during the 8-hour shift: At startup, we loaded all the SurePath vials to be processed that day together with the required consumables and reagents (pipette tips, extraction trays, PCR plates, quality controls [QCs], diluent bottles, M tubes, and reagent troughs), and a second interaction in the afternoon was necessary to replenish pipette tips, extraction trays, M tubes, and PCR plates. The BD COR system has 6 extraction drawers (capacity = 30 patient samples and 2 QCs), 5 of which are replenished in the afternoon to allow the instrument to continue processing unattended, for a combined total of 11 × 30 = 330 specimens. Weekly maintenance was not included in the daily contact time but consisted of cleaning selected touch points on the GX and PX units with 1% bleach, which took approximately 30 minutes.

### Data Analysis

A sample was considered Onclarity positive (for both instruments) if the cycle threshold value was 34.2 or less for HPV-18, 31, 45, 51, 52, 33/58, 35/39/68, and 56/59/66 and 38.3 or less for HPV-16. The GP-EIA detects all 14 HPV genotypes detected by the Onclarity assay as a pooled hrHPV positive result. Between the Onclarity assay on the Viper LT and COR instruments, the level of genotype agreement was determined by the percentage of overall agreement and κ statistics, as was the reproducibility element. The following categories were distinguished based on κ statistics: 0.00-0.20 = poor, 0.21-0.40 = fair, 0.41-0.60 = moderate, 0.61-0.80 = good, and 0.81-1.00 = excellent.^23^

The accuracy of the GP-EIA assay was used as a comparator for clinical validation of the Onclarity assay on the COR instrument. Noninferiority assessment of the Onclarity assay on the COR instrument compared with the GP-EIA assay was according to the international guidelines using the preset 90% and 98% benchmarks for relative sensitivity and specificity, respectively.^2,24^ The reproducibility element was evaluated by using the predefined setup with an 87% or greater lower confidence bound and a κ value above 0.6, as defined by the international criterion.^2^

### Ethical Approval

Sample collection and data retrieval for the VALGENT4 study were approved by the Danish Data Inspection Agency J. No. AHH-2017-024, I-Suite: 05356.

All collected samples were cross-referenced and found eligible with the Danish register on collection, storage, and use of human biological material in health research projects (Vævsanvendelsesregistret).

## Results

### HPV Genotype Test Positivity and Concordance for the Onclarity HPV Assay on the COR and Viper LT Instruments

The overall HPV positivity of Onclarity-Viper and Onclarity-COR was similar, as was the concordance of the 9 individually reported genotypes  Table 1  and  Table 2 . Genotype detection concordance in ≥CIN2 samples showed κ values ranging from good for HPV-51 (κ = 0.76) to excellent for the other 8 genotype groups (κ = 0.90-1.00). Genotype detection concordance for 2×NILM samples showed κ values ranging from good for HPV-51 (κ = 0.80) and HPV-33/58 (κ = 0.78) to excellent for the remaining 7 genotype groups (κ = 0.85-1.00). Considering the entire cohort, the κ values were excellent for all 9 genotype groups (κ = 0.92-0.98)  Table 2 .

**Table 2:** Genotype Distribution and Concordance for Onclarity-COR and Onclarity-Viper, Respectively

HPV Genotypes	COR, No. (%)	Viper LT, No. (%)	COR+/Viper LT+, No.	COR+/Viper LT−, No.	COR−/Viper LT+, No.	COR−/Viper LT−, No.	Agreement, % (95% CI)	κ
≥CIN2 (n = 122)								
16	50 (41.0)	51 (41.8)	50	0	1	71	99.2 (95.5-100)	0.98
18	12 (9.8)	12 (9.8)	12	0	0	110	100 (97.0-100)	1.00
31	18 (14.8)	18 (14.8)	18	0	0	104	100 (97.0-100)	1.00
45	9 (7.4)	10 (8.2)	9	0	1	112	99.2 (95.5-100)	0.94
51	7 (5.7)	6 (4.9)	5	2	1	114	97.5 (93.0-99.5)	0.76
52	19 (15.6)	18 (14.8)	17	2	1	102	97.5 (93.0-99.5)	0.90
33/58	14 (11.5)	14 (11.5)	14	0	0	108	100 (97.0-100)	1.0
35/39/68	6 (4.9)	7 (5.7)	6	0	1	115	99.2 (95.5-100)	0.92
56/59/66	14 (11.5)	13 (10.7)	13	1	0	108	99.2 (95.5-100)	0.96
14 hrHPV	115 (94.3)	114 (93.4)	114	1	0	7	99.2 (95.5-100)	0.93
2×NILM (n = 887)								
16	19 (2.1)	18 (3.0)	17	2	1	867	99.7 (99.0-99.9)	0.92
18	4 (0.5)	4 (0.5)	4	0	0	883	100 (99.6-100)	1.0
31	3 (0.3)	4 (0.5)	3	0	1	883	99.9 (99.4-100)	0.86
45	4 (0.5)	5 (0.6)	4	0	1	882	99.9 (99.4-100)	0.89
51	2 (0.2)	3 (0.3)	2	0	1	884	99.9 (99.4-100)	0.80
52	12 (1.4)	13 (1.5)	12	0	1	874	99.9 (99.4-100)	0.96
33/58	9 (1.0)	9 (1.0)	7	2	2	876	99.5 (98.8-99.9)	0.78
35/39/68	15 (1.7)	18 (2.0)	14	1	4	868	99.4 (98.7-99.8)	0.85
56/59/68	11 (1.2)	11 (1.2)	11	0	0	876	100 (99.6-100)	1.0
14 hrHPV	66 (7.4)	70 (7.9)	62	4	8	813	98.6 (97.6-99.3)	0.90
VALGENT4 panel (n = 1,295)								
16	97 (7.5)	97 (7.5)	94	3	3	1,195	99.5 (99.0-99.8)	0.97
18	30 (2.3)	31 (2.4)	30	0	1	1,264	99.9 (99.6-100)	0.98
31	50 (3.9)	51 (3.9)	48	2	3	1,242	99.6 (99.1-99.9)	0.95
45	33 (2.5)	35 (2.7)	33	0	2	1,260	99.8 (99.4-100)	0.97
51	31 (2.4)	30 (2.4)	28	3	2	1,262	99.6 (99.1-99.9)	0.92
52	47 (3.6)	48 (3.7)	45	2	3	1,245	99.6 (99.1-99.9)	0.95
33/58	46 (3.6)	46 (3.6)	43	3	3	1,246	99.5 (99.0-99.8)	0.93
35/39/68	56 (4.3)	61 (4.7)	54	2	7	1,232	99.3 (98.7-99.7)	0.92
56/59/66	83 (6.4)	82 (6.3)	81	2	1	1,211	99.8 (99.3-98.7)	0.98
14 hrHPV	369 (28.5)	374 (28.9)	362	7	12	914	98.5 (97.7-99.1)	0.96

2×NILM, NILM at baseline and 12-24 months prior; CIN, cervical intraepithelial neoplasia; HPV, human papillomavirus; hrHPV, high-risk human papillomavirus; Onclarity-COR, Onclarity HPV Assay on the COR instrument; Onclarity-Viper, Onclarity HPV Assay on the Viper LT instrument.

### Clinical Performance of Onclarity-COR

The clinical accuracy of Onclarity-COR and the comparator assay (GP-EIA) is shown in  Table 3 . The absolute sensitivity for ≥CIN2 was 94.3% for Onclarity-COR and 92.6% for GP-EIA; the relative sensitivity was 1.02 (95% confidence interval [CI], 0.99-1.04). The absolute sensitivity for ≥CIN3 was 96.4% for Onclarity-COR and 94.0% for GP-EIA; relative sensitivity was 1.03 (95% CI, 0.99-1.06). The Onclarity-COR was found to be noninferior to the comparator assay for both ≥CIN2 (*P* = .0016) and ≥CIN3 (*P* = .0005) sensitivity. The absolute specificity was 92.6% for Onclarity-COR and 89.3% for the comparator assay; relative specificity was 1.04 (95% CI, 1.02-1.05). Onclarity-COR was found to be noninferior to the comparator assay for specificity (*P* < .0001).

**Table 3:** Clinical Accuracy of COR and GP-EIA for ≥CIN2, ≥CIN3, and <CIN1 Outcomes

		GP-EIA Results, No.			Accuracy of COR (95% CI)	Accuracy of GP-EIA (95% CI)	Relative Accuracy (COR/GP-EIA) (95% CI)	Noninferiority Test (*P* Value)
Study Population	COR Results	Pos	Neg	Total				
≥CIN2 (n = 122)	Pos	113	2	115	Sensitivity 94.3% (88.5-97.7)	Sensitivity 92.6% (86.5-96.6)	Sensitivity 1.02 (0.99-1.04)	.0016
	Neg	0	7	7				
	Total	113	9	122				
≥CIN3 (n = 83)	Pos	78	2	80	Sensitivity 96.4% (89.8-99.2)	Sensitivity 94.0% (86.5-98.0)	Sensitivity 1.03 (0.99-1.06)	.0005
	Neg	0	3	3				
	Total	78	5	83				
2×NILM^3^ (n = 887)	Pos	59	7	66	Specificity 92.6% (90.6-94.2)	Specificity 89.3% (87.1-91.2)	Specificity 1.04 (1.02-1.05)	<.0001
	Neg	36	785	821				
	Total	95	792	887				

2×NILM, NILM at baseline and 12-24 months prior; CIN, cervical intraepithelial neoplasia; GP-EIA, glycoprotein 5–positive/6–positive enzyme immunoassay; Neg, negative; Pos, positive.

### Inter- and Intralaboratory Reproducibility

The intralaboratory reproducibility and interlaboratory agreement were assessed using 525 cervical cancer screening samples. The intralaboratory reproducibility of Onclarity-COR was 98.3% (95% CI, 96.8-99.2), with a κ of 0.96  Table 4 . The interlaboratory agreement was 98.5% (95% CI, 97.0-99.3), with a κ of 0.96  Table 4 .

**Table 4:** Intralaboratory Reproducibility and Interlaboratory Agreement

Assessment and Site	HPV Status	Copenhagen Laboratory Result 1, No.		Total, No.	Reproducibility/Agreement, % (95% CI)	κ
		hrHPV Pos	hrHPV Neg			
Intralaboratory reproducibility						
Copenhagen laboratory result 2	hrHPV pos	152	6	158	98.3 (96.8-99.2)	0.96
	hrHPV neg	3	364	367		
	Total	155	370	525		
Interlaboratory agreement						
Østfold laboratory result	hrHPV pos	152	5	157	98.5 (97.0-99.3)	0.96
	hrHPV neg	3	365	368		
	Total	155	370	525		

HPV, human papillomavirus; hrHPV, high-risk human papillomavirus; Neg, negative; Pos, positive.

When looking at the individual genotype concordance  Table 5 , the agreement was excellent for all 9 genotype groups for both the intralaboratory reproducibility (κ = 0.89-1.00) and interlaboratory agreement (κ = 0.93-1.00).

**Table 5:** Intralaboratory Reproducibility and Interlaboratory Agreement of Individual Genotyping With Onclarity-COR

Assessment and HPV type	Concordance per Run/Laboratory, No.				Agreement, % (95% CI)	κ
	Pos/Pos	Pos/Neg	Neg/Pos	Neg/Neg		
Intralaboratory reproducibility (Copenhagen 1/Copenhagen 2)						
HPV-16	49	0	4	472	99.2 (98.1-99.8)	0.96
HPV-18	8	1	0	516	99.8 (98.9-100.0)	0.94
HPV-31	20	2	1	502	99.4 (98.3-99.9)	0.93
HPV-45	7	0	0	518	100 (99.3-100.0)	1.00
HPV-51	8	0	0	517	100 (99.3-100.0)	1.00
HPV-52	11	0	1	513	99.8 (98.9-100.0)	0.96
HPV-33/58	20	2	1	502	99.4 (98.3-99.9)	0.93
HPV-35/39/68	36	3	0	486	99.4 (98.3-99.9)	0.96
HPV-56/59/66	27	3	3	492	98.9 (97.5-99.6)	0.89
Interlaboratory agreement (Copenhagen 1/Østfold)						
HPV-16	47	2	4	472	98.9 (97.5-99.6)	0.93
HPV-18	8	1	0	516	99.8 (98.9-100.0)	0.94
HPV-31	20	2	0	503	99.6 (98.6-100.0)	0.95
HPV-45	7	0	0	518	100 (99.3-100.0)	1.00
HPV-51	8	0	0	517	100 (99.3-100.0)	1.00
HPV-52	11	0	1	513	99.8 (98.9-100.0)	0.96
HPV-33/58	21	1	0	503	99.8 (98.9-100.0)	0.98
HPV-35/39/68	38	1	0	486	99.8 (98.9-100.0)	0.99
HPV-56/59/66	28	2	1	494	99.4 (98.3-99.9)	0.95

HPV, human papillomavirus; Neg, negative; Onclarity-COR, Onclarity HPV Assay on the COR instrument; Pos, positive.

### Time-and-Motion Study

**Figure 1:**
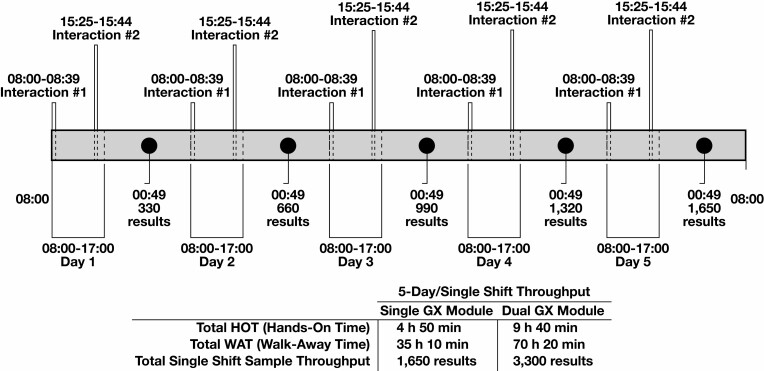
Daily and weekly results output from the COR single–analytical unit (GX) and dual-GX system configurations.

The time-and-motion study was performed to assess the sample turnaround time and overall single-shift production capacity of the COR instrument  Figure 1 . The first user interaction required on average 39 minutes of hands-on time, including daily cleaning, loading of samples and reagents, and unloading samples from the day before. The average walk-away time thereafter was 7 hours, 2 minutes. The second interaction, including loading of reagents and unloading of processed samples and reagents, required on average 19 minutes of hands-on time. Time to result was 4 hours, 15 minutes for the first 30 samples loaded, whereas the successive batches of 30 test results was completed 1 hour, 13 minutes apart  Figure 1 . Using this workflow, the COR system can process 330 samples in 1 8-hour shift, with up to 1,650 reported test results in 1 working week  Figure 1 .

## Discussion

This study presents our validation of the Onclarity HPV assay on the novel high-throughput COR instrument using SurePath collected screening samples from Danish routine cervical cancer screening. Onclarity-COR clinical and analytical performance was similar to Onclarity-Viper on all parameters evaluated  Table 1  and  Table 2 . Onclarity-COR showed noninferior clinical sensitivity (relative sensitivity for ≥CIN2, 1.02 [95% CI, 0.99-1.04]) and noninferior and even slightly higher specificity (relative specificity, 1.04 [95% CI, 1.02-1.05]) compared with GP-EIA  Table 3 . Similar results were found in the previous clinical validation of the Onclarity HPV test on the Viper LT instrument.^14^

Also, the intralaboratory reproducibility and interlaboratory agreement fulfilled the international validation criteria  Table 4 . The intralaboratory reproducibility was 98.3% (κ = 0.96), and the interlaboratory agreement was 98.5% (κ = 0.96) using preprocessed parent laboratory aliquots from the original sample vials. In our previous study evaluating the Onclarity HPV test on the Viper LT instrument,^12^ the interlaboratory agreement was 96.8% (κ = 0.92), and the intralaboratory reproducibility was 97.4% (κ = 0.93). It should be noted, however, that Viper LT and COR testing was performed on 2 different reproducibility panels.

Reproducibility among the individual genotype groups showed excellent concordance for all 9 genotype groups for both intralaboratory reproducibility  Table 5  (κ = 0.89-0.96) and interlaboratory agreement  Table 5  (κ = 0.93-1.00).

Comparing COR performance with similar high-throughput instruments—the Roche cobas 6800/8800 and the Abbott Alinity instruments—inter- and intralaboratory analysis showed equally good results, at more than 98% reproducibility for cobas in the 2 separate studies,^5,6^ and the intra- and interlaboratory reproducibility of the Alinity HPV test^7^ was 96.7% (κ = 0.92) and 98.7% (κ = 0.97), respectively.

The time-and-motion study evaluated a workflow of 330 samples per day and a production of 1,650 test results in a regular week. In comparison, the Viper LT instrument processed 90 to 120 samples per day per instrument.^22^

The average daily hands-on time for a full COR workflow was 58 minutes, with 2 interactions (of 39 minutes and 19 minutes, respectively). The daily walk-away time was 7 hours, 2 minutes  Figure 1 . We used a 1 GX–to–1 PX configuration, but extending the configuration to 2 GX units and 1 PX unit, the COR system can process 660 samples daily and 3,300 test results per regular 5-day week. A limitation in our analysis is the restriction to an 8-hour shift with a “cold start” each day. For high-throughput laboratories, the continuous loading function using the internal sample “hotel” storage of 480 BD SurePath liquid-based cytology vials allows “carry-over” activity between working days but was not assessed in our study. If the system is loaded to capacity with vials on day 1, additional preanalytic processing can be performed overnight, increasing throughput to approximately 500 samples on subsequent days.

A similar approach evaluating the cobas HPV assay on the cobas 6800 instrument showed comparable test turnaround figures,^4^ where initialization, time to first results, time to last result, and total hands-on time amounted to 24 minutes; 2 hours, 28 minutes; 7 hours, 7 minutes; and 59 minutes, respectively. In contrast to our study, the cobas 6800 study did not include preanalytical processing before testing on the cobas 6800 instrument; a recent study estimated that the combined preanalytical (cobas p480) and analytical throughput of the cobas 6800 instrument was 384 samples in an 8-hour shift. To the best of our knowledge, similar time-and-motion studies have not been published on the cobas 8800 or Alinity instruments. Hands-on time, maintenance, and cleaning operations on a high-throughput HPV instrument can greatly affect the daily workload of a laboratory.^3,4,25^

The 3 high-throughput HPV test instruments from Abbott, Becton Dickinson, and Roche, respectively, rely on widely different strategies with respect to reporting HPV-positive findings. HPV genotyping can play a major role in risk management of HPV-positive women, allocating women to risk tiers by HPV genotype^26-28^ and distinguishing new from persistent infections.^29^ The cobas HPV assay maintains the 2010 assay design on all cobas instruments, with individual reporting of HPV-16 and HPV-18 and reporting of the remaining 12 hrHPV genotypes as 1 group.^9^ The Abbott RealTime High Risk HPV Assay on the Alinity instrument is the newest design of the 3 and individually reports HPV genotypes 16, 18, and 45, whereas HPV-31/33/52/58 and HPV-35/39/51/56/59/66/68 are reported in 2 groups.^10^

In comparison, the Onclarity assay allows for extended genotyping on every positive sample, with individual typing of 6 genotypes (HPV-16, 18, 31, 45, 51, 52) and the remaining 8 genotypes in 3 groups (33/58, 35/39/68, 56/59/66). In the United States, Australia, many European Union countries, and other countries, partial genotyping for HPV-16 and HPV-18 is used to risk-stratify women for follow-up.^28,30^ The use of extended genotyping is gaining ground, however, and several studies have shown the 12 non–HPV-16/HPV-18 hr genotypes to carry distinct and markedly different risk of cervical disease.^31-33^ Consequently, more detailed genotyping than just HPV-16 and HPV-18 could be important in near-future triage algorithms and risk management. Of special note for the Onclarity and Alinity assays, the joint probe for HPV-33 and HPV-58 may result in overreferrals because the risk of ≥CIN2 is significantly greater for HPV-33 compared with HPV-58,^26,31,34-36^ and a separation of those 2 genotypes could be beneficial in improving assay specificity.

A major strength of our study is that the Onclarity assay on the COR instrument was validated against the assay on the already-validated Viper LT instrument as well as against an internationally recognized comparator assay, the GP-EIA assay.^2^ In comparison, the novel cobas 6800/8800 HPV assay on the cobas 6800 instrument was validated against the cobas 4800 assay.^5,6^ The argument for this deviation was that the 2 internationally recognized comparator assays—GP-EIA and Hybrid Capture 2 (Qiagen)—have been more or less discontinued in clinical routine worldwide. We acknowledged the merit of this argument, and the guidelines are expected to extend the number of standard comparator tests to reflect this reality.

Another strength of our study is that the COR testing was done using the same panel of samples as previously used to validate the Onclarity assay on the Viper LT instrument. This approach makes the results directly comparable yet also confides a weakness in that the test rounds are 3 years apart. If storage had any negative impact, however, we would have expected performance differences. Such was not observed, and the Onclarity-COR concordance to the comparator assay remained high and well within validation criteria acceptance.

## Conclusion

The Onclarity assay was successfully migrated from the medium-throughput BD Viper LT HPV instrument to the high-throughput BD COR instrument. The Onclarity assay on the novel COR instrument was found to be noninferior to the comparator assay for clinical sensitivity and specificity. The inter- and intralaboratory reproducibility was high. The Onclarity assay allows for extended genotyping, which, together with the COR instrument’s capacity to run a high number of samples, makes the COR instrument an excellent candidate for use in HPV primary cervical cancer screening in laboratories that use screening algorithms, including genotyping.
